# A prescription for physician excellence^☆^

**DOI:** 10.1016/j.bjorl.2016.06.003

**Published:** 2016-07-06

**Authors:** Daniela Carvalho

**Affiliations:** a University of California, Department of Surgery, San Diego, United States; b Rady Children's Hospital of San Diego, Department of Pediatric Otolaryngology, San Diego, United States

The fast pace of innovations in medicine and technology as well as the abundance of information available to doctors and patients is continually reshaping the notion of what constitutes an excellent doctor.

A hundred years ago physicians acquired most of their medical knowledge during their medical school training years. A doctor's professional excellence mainly stemmed from their availability and the degree of attention and care dispensed to the immediate community. Today the stakes have progressively become much higher and a doctor needs to continuously seek new knowledge and rethink old practices. But what exactly do physicians need to do to be considered excellent nowadays? In the next paragraphs we shall attempt to *write a prescription* that can help doctors achieve that goal, with the following ingredients:

- Current best evidence;
- Critical thinking;
- Continued education;
- Collaboration, communication and collegiality;
- De-centralized learning;
- Compassion.

We now live in an era of *Current Evidence-Based Medicine*, in which physicians are encouraged to use the most current evidence found in the literature to make clinical decisions for an individual patient.^1^ But how do we stay current with the ever-growing amount of new evidence published around the world? Learning how to identify trustworthy information can help. One of the ways of doing this is by utilizing *levels of evidence*, where research is arranged in a ranking system to describe the strength of the results measured in the study. Meta analyses and randomized controlled trials are ranked at level 1, the highest level, while case series or expert opinions at the lowest. By using such a scale, the reader can quickly weigh the level evidence of a certain paper in the literature.

It is essential to keep in mind that the designated level of evidence does not always guarantee the quality of the research. One should not assume that *every* level 1 evidence – or that *only* level 1 evidence – is always the best choice for the research question. In otolaryngology, as a surgical specialty, there are many important papers that may have a lower level of evidence (sometimes due to the level of innovation involved, as in a demonstration of a novel surgical technique, for example). Another great tool available for physicians are the *Clinical Practice Guidelines* (CPG), which are created by a group of experts that assess the evidence about important clinical questions or conditions based on systematic reviews. This evidence is then translated into a recommendation within a clinical practice guideline.^2^

All this information is to be used judiciously when treating individual patients with their own specific conditions. This is where *critical thinking* comes into play: a doctor needs to understand patients as a whole (which may include other disorders, cultural aspects and socio-economic status, for example) when using the current literature and CPGs to make clinical decisions.

Another effective way of staying up-to-date with novel information is to engage in events that offer opportunities for *continued education*. Participation in local, national, and society meetings are a fantastic way of keeping current with the latest knowledge and advances and to exchange ideas with peers. Online courses and forums are very helpful as well and can eliminate financial and geographical barriers that can be encountered when traveling to meetings.

Other ingredients included in this excellent doctor's prescription are *collaboration, communication and collegiality*. These come hand in hand, and are crucial for patient care. Collaboration in health care is defined as health care professionals assuming complementary roles and cooperatively working together, sharing responsibility for problem-solving and making decisions to formulate and carry out plans for each patient.^3^ This collaboration needs to happen within our own specialties, between different specialists, and among the various health professionals (doctors, nurses, respiratory therapists, audiologists, etc.). In a hospital setting, collaboration has been shown to decrease errors and improve the quality of care. It works most efficiently if there is collegiality and good communication among health care professionals. Unfortunately these are not stressed enough during medical school, as physicians are taught to “know it all” and “do it all”, and often forget how important these ingredients are to deliver exceptional care to our patients.

In regards to our medical education, great part of it comes in the form of lessons received from teachers during medical school, residency and beyond. Besides the technical aspects of medicine, each teacher and mentor influences us with their different ways of relating to patients, subtle cultural nuances, and distinctive thought processes. In the surgical field we also relate to the particular ways of someone performing surgical procedures. For many disease processes there is no single *correct* answer, so learning something in different ways (for example, different surgical techniques for a certain condition) can allow an individual to determine what works best in a physician's practice and for a specific patient. In the United States it is very common for physicians to go to college, medical school, residency and fellowship in different institutions. By doing so future doctors are exposed to more teachers and different “medical cultures”, which can enhance their education as physicians. In places where most trainees continue in their own institution there might be a bias in the selection of future candidates and less exchange of information. This is another reason why a *decentralized learning process* is important in the medical education. But one doesn’t necessarily need to travel far or spend a long time in a different institution to accomplish that. Visiting other centers, even if in the same town or state (and even for a short period of time), can be very beneficial.

Even the most knowledgeable and up-to-date physician with the best technical skills will not become excellent without *compassion*. In spite of all the knowledge and technology available today, being able to respect and listen to our patients and to treat them with the dignity and care that they deserve continues to be, as it did a hundred years ago, a crucial part of what constitutes being an outstanding doctor.

We have chosen a challenging but much rewarding profession. Being an excellent doctor requires continued balance between the knowledge and the art of medicine. This *prescription for physician excellence* should be taken daily. Long-term side effects include a rewarding professional life and a host of grateful patients, families and co-workers.

## Conflicts of interest

The author declares no conflicts of interest.
